# Sociodemographic characteristics and diabetes predict invalid self-reported non-smoking in a population-based study of U.S. adults

**DOI:** 10.1186/1471-2458-7-33

**Published:** 2007-03-12

**Authors:** Monica A Fisher, George W Taylor, Brent J Shelton, Sara M Debanne

**Affiliations:** 1 Case Western Reserve University, Cleveland, OH, USA; 2 University of Michigan, Ann Arbor, MI, USA; 3 University of Kentucky, Lexington, KY, USA

## Abstract

**Background:**

Nearly all studies reporting smoking status collect self-reported data. The objective of this study was to assess sociodemographic characteristics and selected, common smoking-related diseases as predictors of invalid reporting of non-smoking. Valid self-reported smoking may be related to the degree to which smoking is a behavior that is not tolerated by the smoker's social group.

**Methods:**

True smoking was defined as having serum cotinine of 15+ng/ml. 1483 "true" smokers 45+ years of age with self-reported smoking and serum cotinine data from the Mobile Examination Center were identified in the third National Health and Nutrition Examination Survey. Invalid non-smoking was defined as "true" smokers self-reporting non-smoking. To assess predictors of invalid self-reported non-smoking, odds ratios (OR) and 95% confidence intervals (CI) were calculated for age, race/ethnicity-gender categories, education, income, diabetes, hypertension, and myocardial infarction. Multiple logistic regression modeling took into account the complex survey design and sample weights.

**Results:**

Among smokers with diabetes, invalid non-smoking status was 15%, ranging from 0% for Mexican-American (MA) males to 22%–25% for Non-Hispanic White (NHW) males and Non-Hispanic Black (NHB) females. Among smokers without diabetes, invalid non-smoking status was 5%, ranging from 3% for MA females to 10% for NHB females. After simultaneously taking into account diabetes, education, race/ethnicity and gender, smokers with diabetes (OR_Adj _= 3.15; 95% CI: 1.35–7.34), who did not graduate from high school (OR_Adj _= 2.05; 95% CI: 1.30–3.22) and who were NHB females (OR_Adj _= 5.12; 95% CI: 1.41–18.58) were more likely to self-report as non-smokers than smokers without diabetes, who were high school graduates, and MA females, respectively. Having a history of myocardial infarction or hypertension did not predict invalid reporting of non-smoking.

**Conclusion:**

Validity of self-reported non-smoking may be related to the relatively slowly progressing chronic nature of diabetes, in contrast with the acute event of myocardial infarction which could be considered a more serious, major life changing event. These data also raise questions regarding the possible role of societal desirability in the validity of self-reported non-smoking, especially among smokers with diabetes, who did not graduate from high school, and who were NHB females.

## Background

Smoking is a major risk factor for many diseases [1], including highly prevalent conditions such as heart disease [1-3], diabetes and its complications [1,3-14], and hypertension [15,16]. According to the Standards of Medical Care in Diabetes, hypertension is a common comorbidity of diabetes, "*In these patients, other cardiovascular risk factors including obesity, hyperlidemia, smoking ... should be carefully assessed and treated*." [17]. These standards of care support smoking cessation counseling as a necessary component of comprehensive approaches to manage patients with diabetes because smoking is related to macrovascular and microvascular complications of diabetes [17]. However, neither health care providers nor individuals with diabetes are sufficiently aware of the increased risk of developing cardiovascular disease for smokers with diabetes [18,19]. Preventive care to reduce the risk of future cardiac events includes interventions targeting type 2 diabetes, obesity and insulin resistance such as the use of medications and lifestyle changes to stop smoking, increase exercise, and incorporate diet modification to lower blood glucose levels, blood pressure, and cholesterol [17,20-24]. Health care providers' compliance with diabetes-related preventive care has been low [25].

While smoking status is routinely measured through self-report, the validity of such self-reported smoking data may be suspect because individuals may give invalid self-report of their smoking status. Carbon monoxide and nicotine/cotinine are widely used biomarkers of tobacco or tobacco smoke exposure providing objective measures of smoking data [26-32]. Nearly all studies reporting smoking status collect self-reported data that are very rarely validated. For example, there were 191 original articles, clinical practice, clinical implications of basic research and health policy reports with "smoking" in the text of *the New England Journal of Medicine *from January 2001 through December 2005, of which 1.0% (n = 2) included cotinine-determined smoking measures.

Self-reported smoking status underestimates smoking prevalence in certain populations [33-35]. Data relevant to identifying smokers who deny smoking in a meta-analysis on validity of self-reported smoking status reported an average 13% of "true" smokers reported invalid non-smoking status, with a wide variation ranging from 0% to 94% [36]. This wide variation may be related to the different study designs including distinct objective measures of smoking such as carbon monoxide, carboxyhemoglobin, thiocyanate, serum or salivary cotinine, and very different study populations.

One important question is whether the proportion of respondents who smoke but report they are not smoking is a systematic (i.e. differential) misclassification bias so as to confound the relationships between predictors and dependent variables. Smoking misclassification bias has been found with a correlation as low as 0.40 between self-reported and cotinine-determined smoking [31]. Invalid reporting of smoking may explain the effect of a main exposure (eg. beta-carotene), indicating the need to include biomarkers of smoking in epidemiologic studies [31]. In other words, if smoking is measured with error, and smoking is associated with an exposure of interest, it will be difficult to rule out smoking as the true cause of the association between the exposure of interest and the outcome, hence leading to a biased estimate.

When looking at the evidence, with respect to significant proportions of smokers who self-reported as non-smokers in population-based studies, it may be expected that the more social pressure exists against smoking in a society or the personally relevant social network of the smoker, the larger may be her/his propensity to deny smoking. It is hypothesized that people who have or have had smoking-related severe diseases such as diabetes or myocardial infarction have a strong propensity to deny smoking. Thus, there are sociodemographic characteristics such as age, race/ethnicity, gender, education and income, along with smoking-related diseases in which patients are advised not to smoke, that may be of relevance for invalid self-reported statements.

The current literature supports a recommendation that self-reported smoking status be confirmed by biochemical verification [26-32,37]. Descriptive studies found many patients deny smoking [38], even after having been informed that they would be tested for tobacco smoke exposure [39]. A research approach referred to as the "bogus pipeline" strategy, informs study participants that their self-reports can or will be verified by a monitoring device [40]. As applied to self-reported tobacco use, participants are informed that their self-reports can or will be verified by a biochemical test, then specimens are collected but not analyzed. This approach may not be relevant to the third National Health and Nutrition Examination Survey (NHANES III) participants because they were aware that their serum would be tested for many components, but there was not a focus on the cotinine assay.

Typically, studies reporting on validity of self-reported smoking for different groups have used a single covariate, such as, race [41,42], age [43,44], gender [44], social class [44], marital status [44], education [44]; or race-gender categories [45]. While the question of the accuracy of self-reported smoking has been previously reported, this question has not been approached through a population-based study simultaneously assessing age, race/ethnicity-gender, education, income, and smoking-related diseases as predictors of invalid self-reported non-smoking by "true" smokers. Previous studies focused on self-reported smoking as the denominator, not on "true" smokers. That is, previous studies investigated the question, among those who report they smoke or do not smoke, what proportion truly smoke or do not smoke. But, there has not been a focus on the question, among those who truly smoke, what proportion report they do not smoke.

The objectives of our study include: (1) Describe sociodemographic characteristics and smoking-related disease status for valid and invalid reporting of smoking status by "true" smokers; and (2) Assess predictors by quantifying the association between invalid self-reported non-smoking and age, race/ethnicity, gender, income, education, diabetes, myocardial infarction, and hypertension, among "true" smokers in NHANES III [46].

## Methods

### Study population

The third NHANES, 1988–1994 is a national examination survey of civilian noninstitutionalized individuals, representative of the US population. NHANES employs a complex, multistage, stratified, clustered sample design. NHANES includes questionnaire, laboratory assays and clinical examination measures of health outcomes and explanatory variables. The data relevant to this study are: responses to questions regarding age, race/ethnicity, gender, household income, education, history of tobacco use, diabetes, myocardial infarction, hypertension, and laboratory assay of serum cotinine. This study was reviewed by the Case Western Reserve University institutional review board and approved as exempt under 45 Code of Federal Regulations part 46.101b, #4, IRB Protocol Number 20050805. In addition, all investigators complied with the Data Use Restrictions for the NHANES III public-use data set.

Participants provided informed consent to voluntarily participate in the interview, or the interview and examination, or the interview, examination and laboratory tests [46]. We identified 7295 adults, ages 45+ years, who completed the Household Adult Questionnaire indicating they did not currently use smokeless tobacco, pipe or cigars, and completed the Mobile Examination Center (MEC) question "How many cigarettes have you smoked in the past 5 days?" and reported they did not use nicotine gum in the past 5 days. Among the 7295 participants with MEC smoking status 410 (4.3% weighted percent) did not have serum cotinine data, resulting in 6885 adults, ages 45+ years, who did not use smokeless tobacco, pipe, cigars, or nicotine gum with MEC smoking status and MEC serum cotinine data. Thus, we studied 1483 (22.0% weighted percent) cotinine-determined smokers 45+ years of age in the NHANES III data set who did not currently use smokeless tobacco, pipe, cigars, or nicotine gum. We restricted our analyses to 45+ year olds because studies investigating diabetes and myocardial infarction typically assess individuals who develop the condition at this age [5-15].

### Definition of main outcome

The main outcome was invalid non-smoking status, defined as "true" smokers who self-reported as non-smokers during the MEC component of NHANES III. Cotinine is a biochemical measure of tobacco exposure, it is a metabolite of nicotine indicating exposure during the previous 1 to 2 days [29]. Serum cotinine was assayed using an isotope dilution, liquid chromatography, tandem mass spectrometry method with a limit of detection of 0.03 ng/ml [46]. Thus, we used this gold standard to identify "true" smokers as those adults with serum cotinine levels 15+ng/ml [29,47]. The definition of cotinine-determined smokers was based on the finding that serum cotinine level has a bimodal distribution, with a separation between the two peaks at serum cotinine level of 10–15 ng/ml. This distinguishes active tobacco use and secondhand smoke exposure because the highest serum cotinine level in a nonsmoker exposed to second hand smoke is 10–13 ng/ml [30]. Thus, we used the more conservative cutpoint of 15 ng/ml of serum cotinine to identify cotinine-determined smokers who self-reported they currently do not smoke. Henceforth, we will refer to cotinine-determined smokers as "true" smokers.

### Definition of explanatory variables

To meet the objectives of our study we assessed the role that smoking-related diseases may play in the validity of self-reported smoking status by focusing on three highly prevalent smoking-related diseases. We also assessed sociodemographic characteristics that play a role in disease status and may play a role in the amount of social pressure against smoking in the individual's social network. Thus, the potential explanatory variables for invalid self-reported non-smoking status by "true" smokers were age, race/ethnicity, gender, household income, education, diabetes, myocardial infarction, and hypertension status. Age was dichotomized as 45–64 versus 65+ years of age using categories previously used for NHANES publications [48], with six race/ethnicity-gender categories, namely Non-Hispanic White (NHW) females, Non-Hispanic Black (NHB) females, Mexican-American (MA) females, NHW males, NHB males, and MA males. The race/ethnicity category "other" was excluded due to small or zero cell counts. Household income and education were dichotomized as categories previously used in NHANES publications, such that household income was defined as less than $20,000 versus at least $20,000; and education was dichotomized as high school graduate (Yes/No) [49,50].

To further elucidate potential explanatory variables, we assessed three highly prevalent smoking-related diseases in which patients are advised not to smoke, namely diabetes, myocardial infarction, and hypertension. While we hypothesized that people who suffer from these smoking-related diseases may have a strong propensity to deny smoking, we recognize the current clinical guidelines recommend that all patients who use tobacco be advised to quit [51], not just those who already have disease. For males, diabetes status was based on their response to whether they were ever told by a doctor that they had diabetes or sugar diabetes (Yes/No). Females were defined as having diabetes if they had been told by a doctor that they had diabetes or sugar diabetes when they were not pregnant. Women with diabetes only during pregnancy were defined as not having diabetes. Participants responding that a doctor ever told them that they had a heart attack were defined as having a history of myocardial infarction, and those responding that they were told by a doctor or other health professional that they had hypertension, on two or more different visits were defined as having hypertension.

### Statistical analyses

We compared invalid self-reported non-smoking among age, race/ethnicity-gender categories, education, and household income for "true" smokers with and without diabetes, history of myocardial infarction, and hypertension. This study compared two distinct measures of smoking status, self-reported questionnaire data and laboratory assay results for serum cotinine. SAS-callable SUDAAN [52] was used for all analyses, to account for complex survey design and sample weights in NHANES III. To assess predictors of invalid self-reported non-smoking we quantified the association between invalid self-reported non-smoking and age, race/ethnicity-gender categories (reported to indicate interaction), income, education, diabetes, history of myocardial infarction, and hypertension by calculating the odds ratios (OR) and 95% confidence interval (CI) for both the unadjusted or crude OR (OR_Crude_), and the adjusted OR (OR_Adj_) using multiple logistic regression modeling, simultaneously adjusting for the potential explanatory variables.

## Results

### Overall descriptive summary

The descriptive summary for invalid self-reported non-smoking by "true" smokers, who do not currently use smokeless tobacco, pipe, cigars or nicotine gum is reported in Table 1, along with the OR_Crude _for the association between invalid self-reported non-smoking and the potential explanatory variables. The overall invalid reported non-smoking was 5.8%. Older "true" smokers 65+years old (12.6%) were almost 4 times (OR_Crude _= 3.88, 95% CI: 1.95–7.73) more likely to report invalid non-smoking than 45–64 year olds (3.6%). NHB smokers (8.5%) were 1 1/2 times (OR_Crude _= 1.53; 95% CI: 1.04–2.26) more likely to report invalid non-smoking than NHW smokers (5.8%). Smokers who did not graduate from high school (8.6%) were twice as likely (OR_Crude _= 2.04; 95% CI: 1.35–3.08) to report non-smoking as high school graduates (4.4%). Smokers with diabetes (15.0%) were over 3 times (OR_Crude _= 3.26; 95% CI: 1.38–7.72) more likely to report non-smoking than smokers without diabetes (5.1%). There was no statistically significant association between invalid self-reported non-smoking and gender, income, history of myocardial infarction, or hypertension.

**Table 1:** Descriptive summary and association with validity of self-reported smoking status, 45+ year old "true" smokers, United States, 1988–1994.

	Invalid Self-Report		Valid Self-Report		
			
Explanatory Variables	112 (n)	5.8%	1371 (n)	94.2%	OR_Crude _95% CI
Gender					
Female	52	5.7%	614	94.3%	1.00
Male	60	5.9%	757	94.1%	1.05 (0.65–1.70)
Age (years old)					
45–64	48	3.6%	962	96.4%	1.00
65+	64	12.6%	409	87.4%	3.88 (1.95–7.73)*
Race/Ethnicity					
Non-Hispanic White	48	5.8%	615	94.2%	1.00
Non-Hispanic Black	48	8.5%	427	91.5%	1.53 (1.04–2.26)*
Mexican-American	15	5.1%	279	94.9%	0.87 (0.42–1.81)
Income					
Less than $20,000	69	6.6%	738	93.4%	1.31 (0.74–2.33)
$20,000 or more	39	5.1%	602	94.9%	1.00
High School Graduate					
Yes	39	4.4%	671	95.6%	1.00
No	73	8.6%	688	91.4%	2.04 (1.35–3.08)*
Diabetes					
Yes	19	15.0%	118	85.0%	3.26 (1.38–7.72)*
No	93	5.1%	1251	94.9%	1.00
Myocardial Infarction					
Yes	10	6.4%	97	93.6%	1.12 (0.42–2.99)
No	102	5.8%	1264	94.2%	1.00
Hypertension					
Yes	42	7.0%	395	93.0%	1.35 (0.59–3.08)
No	69	5.3%	970	94.7%	1.00

Note, unweighted number with weighted percent. Bold font identifies statistically significant findings (*p < 0.05).

All adults did not currently use smokeless tobacco, pipe, cigars, or nicotine gum and had serum cotinine data.

"True" smokers were defined as having 15+ng/ml serum cotinine. Invalid self-report was defined as "true" smokers self-reporting as non-smokers. Valid self-report was defined as "true" smokers self-reporting as smokers.

OR_Crude _= Unadjusted odds ratio for the association between invalid self-report and the potential explanatory variable.

### Invalid self-reported non-smoking by "true" smokers with and without diabetes

The age-specific descriptive summary of "true" smokers self-reporting their smoking status is reported in Table 2, along with six gender-race/ethnicity specific categories, stratified by diabetes status. The proportion of "true" smokers with diabetes reporting non-smoking was higher for 65+ year olds (19.8%) than for 45–64 year olds (12.3%). Among 45+ year old smokers with diabetes, 15.0% self-reported as non-smokers, ranging from 0.0% of MA males, to 21.5% of NHW males and 25.4% of NHB females. Among "true" smokers without diabetes the proportion self-reporting invalid non-smoking was higher for older adults, with 3.0% of 45–64 year olds compared to 11.9% of 65+ year olds. Among 45+ year old smokers without diabetes, 5.1% self-reported as non-smokers, ranging from 2.5% of MA females, to 6.8% of MA males and 9.5% of NHB females.

**Table 2:** Number (unweighted) and percent (weighted) of invalid self-reported non-smoking by "true" smokers by age, gender, race/ethnicity and disease status, United States, 45+ year olds, 1988–1994

TRUE SMOKERS WITH DIABETES												
	Age-specific											
							
	45+ year olds		45–64 years old		65+ years old							
	CD smoker		CD smoker		CD smoker							
									
SR smoker	119	85.0%	72	87.7%	46	80.2%						
SR nonsmoker	**19**	**15.0%**	**8**	**12.3%**	**11**	**19.8%**						

	Gender and race-specific											
	
	NHW females		NHB females		MA females		NHW males		NHB males		MA males	
	CD smoker		CD smoker		CD smoker		CD smoker		CD smoker		CD smoker	
						
SR smoker	20	87.7%	24	74.6%	12	92.4%	15	78.5%	22	94.2%	21	100.0%
SR nonsmoker	**3**	**12.3%**	**7**	**25.4%**	**1**	**7.6%**	**5**	**21.5%**	**3**	**5.8%**	**0**	**0.0%**

TRUE SMOKERS WITHOUT DIABETES												

	Age-specific											
							
	45+ year olds		45–64 years old		65+ years old							
	CD smoker		CD smoker		CD smoker							
									
SR smoker	1251	94.9%	889	97.0%	362	88.1%						
SR nonsmoker	**93**	**5.1%**	**40**	**3.0%**	**53**	**11.9%**						

	Gender and race-specific											
	
	NHW females		NHB females		MA females		NHW males		NHB males		MA males	
	CD smoker		CD smoker		CD smoker		CD smoker		CD smoker		CD smoker	
						
SR smoker	297	95.5%	160	90.5%	78	97.5%	282	94.3%	221	94.7%	167	93.2%
SR nonsmoker	**16**	**4.5%**	**21**	**9.5%**	**3**	**2.5%**	**24**	**5.7%**	**17**	**5.3%**	**11**	**6.8%**

TRUE SMOKERS WITH MYOCARDIAL INFARCTION												

	Age-specific											
							
	45+ year olds		45–64 years old		65+ years old							
	CD smoker		CD smoker		CD smoker							
									
SR smoker	97	93.6%	55	98.1%	42	84.4%						
SR nonsmoker	**10**	**6.4%**	**4**	**1.9%**	**6**	**15.6%**						

	Gender and race-specific											
	
	NHW females		NHB females		MA females		NHW males		NHB males		MA males	
	CD smoker		CD smoker		CD smoker		CD smoker		CD smoker		CD smoker	
						
SR smoker	18	93.4%	13	86.0%	3	100.0%	35	94.2%	17	91.9%	7	63.0%
SR nonsmoker	**1**	**6.6%**	**2**	**14.0%**	**0**	**0.0%**	**4**	**5.8%**	**2**	**8.1%**	**1**	**37.0%**

TRUE SMOKERS WITHOUT MYOCARDIAL INFARCTION												

	Age-specific											
							
	45+ year olds		45–64 years old		65+ years old							
	CD smoker		CD smoker		CD smoker							
									
SR smoker	1264	94.2%	898	96.3%	366	87.6%						
SR nonsmoker	**102**	**5.8%**	**44**	**3.7%**	**58**	**12.4%**						

	Gender and race-specific											
	
	NHW females		NHB females		MA females		NHW males		NHB males		MA males	
	CD smoker		CD smoker		CD smoker		CD smoker		CD smoker		CD smoker	
						
SR smoker	298	95.0%	166	88.2%	85	96.7%	262	93.4%	225	94.8%	182	95.8%
SR nonsmoker	**18**	**5.0%**	**26**	**11.8%**	**4**	**3.3%**	**25**	**6.6%**	**18**	**5.2%**	**10**	**4.2%**


TRUE SMOKERS WITH HYPERTENSION												

	Age-specific											
							
	45+ year olds		45–64 years old		65+ years old							
	CD smoker		CD smoker		CD smoker							
									
SR smoker	395	93.0%	263	94.8%	132	88.4%						
SR nonsmoker	**42**	**7.0%**	**19**	**5.2%**	**23**	**11.6%**						

	Gender and race-specific											
	
	NHW females		NHB females		MA females		NHW males		NHB males		MA males	
	CD smoker		CD smoker		CD smoker		CD smoker		CD smoker		CD smoker	
						
SR smoker	92	91.9%	78	87.3%	18	97.1%	80	94.5%	84	93.8%	33	84.4%
SR nonsmoker	**8**	**8.1%**	**13**	**12.7%**	**1**	**2.9%**	**9**	**5.5%**	**8**	**6.2%**	**3**	**15.6%**

TRUE SMOKERS WITHOUT HYPERTENSION												

	Age-specific											
							
	45+ year olds		45–64 years old		65+ years old							
	CD smoker		CD smoker		CD smoker							
									
SR smoker	970	94.7%	695	97.0%	275	86.8%						
SR nonsmoker	**69**	**5.3%**	**28**	**3.0%**	**41**	**13.2%**						

	Gender and race-specific											
	
	NHW females		NHB females		MA females		NHW males		NHB males		MA males	
	CD smoker		CD smoker		CD smoker		CD smoker		CD smoker		CD smoker	
						
SR smoker	225	96.1%	105	89.0%	72	96.8%	218	93.1%	158	95.1%	152	95.7%
SR nonsmoker	**11**	**3.9%**	**14**	**11.0%**	**3**	**3.2%**	**20**	**6.9%**	**12**	**4.9%**	**8**	**4.3%**

Note, unweighted number with weighted percent. Bold font identifies the unweighted number and weighted percent of "true" smokers self-reporting as non-smokers.

All adults did not currently use smokeless tobacco, pipe, cigars, or nicotine gum, and had serum cotinine data.

"True" smokers were defined as having 15+ng/ml serum cotinine. Invalid self-report was defined as "true" smokers self-reporting as non-smokers. Valid self-report was defined as "true" smokers self-reporting as smokers.

SR: self-reported, CD: cotinine-determined; NHW = Non-Hispanic White; NHB = Non-Hispanic Black; MA = Mexican-American

### Invalid self-reported non-smoking by "true" smokers with and without a history of myocardial infarction

In Table 2, the proportion of "true" smokers with a history of myocardial infarction reporting non-smoking was higher for 65+ year olds (15.6%) than for 45–64 year olds (1.9%). Among 45+ year old "true" smokers with a history of myocardial infarction, 6.4% self-reported as non-smokers, ranging from 0.0% of MA females, to 37.0% of MA males. Among "true" smokers without a history of myocardial infarction the proportion self-reporting invalid non-smoking was higher for 65+ year olds (12.4%) than for 45–64 year olds (3.7%). Among 45+ year old "true" smokers without a history of myocardial infarction, 5.8% self-reported as non-smokers, ranging from 3.3%–6.6% of MA females, MA males, NHW females, NHB males and NHW males, to 11.8% of NHB females.

### Invalid self-reported non-smoking by "true" smokers with and without hypertension

In Table 2, the proportion of "true" smokers with hypertension reporting non-smoking was higher among older smokers with 11.6% of 65+ year olds compared to 5.2% of 45–64 year olds. Among 45+ year old true "smokers" with hypertension, 7.0% self-reported as non-smokers, ranging from 2.9% of MA females, to 15.6% of MA males. Among "true" smokers without hypertension the proportion self-reporting invalid non-smoking was higher among older smokers with 13.2% of 65+ year olds compared to 3.0% of 45–64 year olds. Among 45+ year old "true" smokers without hypertension, 5.3% self-reported as non-smokers, ranging from 3.2%–4.9% of MA females, NHW females, MA males, and NHB males, to 11.0% of NHB females.

### Association between invalid self-reported non-smoking and diabetes, history of myocardial infarction, hypertension, and socioeconomic status

The most parsimonious model for the association between invalid self-reported non-smoking and diabetes, simultaneously adjusting for education, and race/ethnicity-gender is reported in Table 3. Among "true" smokers, those with diabetes (OR_Adj _= 3.15; 95% CI: 1.35–7.34) and NHB females (OR_Adj _= 5.12; 95% CI: 1.41–18.58) were 3 to 5 times more likely to report non-smoking than those without diabetes, and MA females, respectively. Among "true" smokers, those who did not graduate from high school (OR_Adj _= 2.05; 95% CI: 1.30–3.22) and NHB females (OR_Adj _= 1.96; 95% CI: 1.17–3.28) were twice as likely to report non-smoking as high school graduates and NHW females, respectively. When we tested a full-model simultaneously adjusting for all three smoking-related diseases, this did not change any conclusions. In the full model the point-estimate for diabetes was essentially unchanged (OR_Adj _= 3.13; 95% CI: 1.33–7.35) and history of myocardial infarction (P = 0.97) and hypertension (P = 0.71) were non-significant variables; therefore they were excluded in the final model.

**Table 3:** Association between invalid self-reported non-smoking and diabetes, education, and race/ethnicity-gender, 45+ year olds, United States, 1988–1994

Explanatory Variable	OR_Adj _95%CI	p-value
Diabetes vs No Diabetes	3.15 (1.35–7.34)*	**0.01**
Not HS Graduate vs HS Graduate	2.05 (1.30–3.22)*	**<0.01**
NHB Females vs NHW Females	1.96 (1.17–3.28)*	**0.01**
NHW Females vs MA Females	2.62 (0.74–9.29)	0.13
NHW Males vs NHW Females	1.31 (0.77–2.23)	0.32
NHB Females vs MA Females	5.12 (1.41–18.58)*	**0.01**
NHB Females vs NHB Males	2.38 (0.93–6.09)	0.07
MA Males vs MA Females	2.24 (0.42–11.99)	0.34
NHW Males vs NHB Males	1.59 (0.76–3.33)	0.21
NHW Males vs MA Males	1.52 (0.56–4.18)	0.41
MA Males vs NHB Males	1.04 (0.36–3.01)	0.94

Bold font identifies the statistically significant associations (* p < 0.05).

Note, all adults did not currently use smokeless tobacco, pipe, cigars, or nicotine gum, and had serum cotinine data. Invalid self-report was defined as "true" smokers self-reporting as non-smokers. Valid self-report was defined as "true" smokers self-reporting as smokers.

HS: High School; NHW: Non-Hispanic White; NHB: Non-Hispanic-Black: MA: Mexican-American

## Discussion

Invalid self-reported non-smoking by "true" smokers is a critical aspect for the clinical management of smokers, when estimating smoking prevalence, estimating excess morbidity and mortality associated with smoking, and when collecting smoking data in clinical trials and surveys and other observational studies. Our study found "true" smokers with diabetes were more likely to report invalid non-smoking than "true" smokers without diabetes, but having a history of myocardial infarction or hypertension did not predict invalid reporting of non-smoking. A possible explanation for these different results regarding predictors of invalid self-reported non-smoking may be related to the relatively slowly progressing chronic nature of diabetes compared to acute myocardial infarction which could be considered a more serious, major life changing event. This possibility is based on the finding that smokers who had a history of myocardial infarction were over four times more likely to quit smoking than those being diagnosed with diabetes [53], and that duration of diabetes was not associated with smoking [54]. Thus smokers who had a history of myocardial infarction may be more likely to truly quit smoking than smokers with diabetes or hypertension.

A possible explanation for our results differing from previous reports may be related to our modeling approach. We assessed invalid self-reported non-smoking by "true" smokers as the outcome, fitting diabetes, education, race/ethnicity and gender main effects along with an interaction term, whereas the other approaches were based on unadjusted analyses or multiple regression analyses of self-reported smoking without interaction terms. The incorporation of an interaction term for race/ethnicity and gender allows for the ascertainment of "true" smokers who are more likely to report non-smoking, in order to develop a targeted approach to identify smokers for smoking cessation interventions. This is important in light of the findings that health care providers and individuals with diabetes are not sufficiently aware of the increased risk of developing cardiovascular disease for smokers with diabetes [18,19].

To the best of our knowledge our study is the first population-based report on invalid self-reported non-smoking restricted to those who do not currently use smokeless tobacco, pipe, cigars, or nicotine gum. Even though there may be some invalid responses to the use of non-cigarette tobacco products, these products were used much less frequently in NHANES III with 4990 current cigarette smokers compared to 602 current smokeless tobacco users, 296 current cigar users and 148 current pipe users. If the proportion of adults who report invalid non-tobacco use is similar for each tobacco group, cigarette users would be impacted the most because of the higher prevalence. This may explain why our results differ from previous reports. Due to this restriction, our study had limited statistical power related to the small sample size in the age, race/ethnicity-gender specific subgroup analyses, especially when the analyses were stratified by diabetes, history of myocardial infarction, and hypertension history. While the oldest (*i.e*., 65+ year old) "true" smokers were more likely to self-report non-smoking than 44–64 year olds, the effect of age was not assessed further due to the small sample size of younger smokers with diabetes or history of myocardial infarction who self-reported as non-smokers.

Deception may explain most of the invalid self-reports of non-smoking [55]. Social desirability related to the social unacceptability of cigarette smoking [56] is an additional possible explanation of the invalid self-reported non-smoking status by "true" smokers. That is, NHB female smokers, smokers with diabetes or who did not graduate from high school may be more prone to being influenced by social pressure to not smoke and thus provide the socially desirable non-smoking response.

In addition, other researchers have investigated potential explanations for serum cotinine levels in non-smokers, including dietary intake of food items previously reported to have measurable levels of nicotine, such as potatoes, tomatoes, eggplant, cauliflower, green peppers, iced tea, and brewed tea [29]. These food items were not significant in the regression models for adults [29].

We believe this limitation is outweighed by the following strengths of our study: 1) focusing on the most relevant patient management, public health surveillance, and study design issues of "true" smokers by incorporating an objective measure to complement self-report; 2) analyses restricted to those who did not currently use smokeless tobacco, cigars, pipes, or nicotine gum addressed the limitation of cotinine being elevated in users of snuff and chewing tobacco [36]; 3) our modeling approach included race/ethnicity-gender interaction; 4) NHANES III sampling methodology is designed to represent the US population; 5) high quality and quantity of questionnaire, examination and laboratory data collected in an unbiased manner such that the participants were unaware of our study on predictors of invalid self-reporting of non-smoking status.

"True" smokers reporting non-smoking have higher mortality rates, higher prevalence of tobacco-related cancer, and higher prevalence of a history of myocardial infarction compared to true nonsmokers [43]. Our findings indicate that the impact of invalid self-reported non-smoking on morbidity and mortality may slightly under-estimate the true impact for 45–64 year olds, with a more substantially under-estimate for 65+ year olds. In addition, the *Healthy People 2010 *objective 27-1a to reduce smoking prevalence [57,58] is based on self-reported data that are generally reported to be valid [36]. However, apparently not considered was the wide variability in the proportion of smokers reporting they were not smokers ranging from 0% to 94% [36]. Our findings raise concerns regarding invalid self-reported non-smoking by specific age, race/ethnicity-gender, education or diabetes. Additional research using a similar approach with larger sample sizes, that simultaneously takes into account sociodemographic and health status characteristics is needed to investigate this study's findings further.

These findings have potentially important general implications. An objective assessment of tobacco use, either through a targeted approach by health care providers, or as the general protocol when collecting tobacco use data in a population surveillance, clinical, or epidemiologic study, identifies smokers who deny smoking. Three general implications are: 1) Identification of smokers who deny smoking provides an opportunity for health care providers to present tobacco cessation counseling to those individuals who would not otherwise receive such advice, with the objective being the improvement of the individual patient's health. 2) Collection of an objective measure of current tobacco use may provide a better estimate of the true prevalence rates in the population and/or among the specific age, race, and gender subgroups. 3) Using an objective measure of tobacco use in a clinical or epidemiologic study addresses the possible explanation that the results may be due to systematic misclassification bias of self-reported tobacco use.

One approach to address smoking misclassification in clinical or epidemiologic studies would be the development of a statistical approach, similar to those available but limited to a single covariate [59,60]. Alternative approaches include incorporating objective smoking measures to complement self-report by excluding "true" smokers who report never, or former tobacco use, or to create and assess a separate category of "true" smokers who report never or former tobacco use. Even though objective tobacco use measures identify current "true" tobacco-users, and most smoking-related diseases are chronic requiring a smoking history, by excluding "true" smokers self-reporting non-smoking (*i.e*., never or former smoking) this decreases smoking misclassification bias, and may decrease bias associated with other inaccurate responses such as self-reported disease status. Further investigation of questions assessing the validity of self-reported smoking status may also be useful.

## Conclusion

We found non-random or systematic bias, indicating the inability to determine the direction in which reported estimates differ from the true effects (under-estimate, over-estimate, or true estimate). Validity of self-reported non-smoking may be related to the relatively slowly progressing chronic nature of diabetes, in contrast with the acute event of myocardial infarction which could be considered a more serious, major life changing event. These data also raise questions regarding the possible role of societal desirability in the validity of self-reported non-smoking, especially among "true" smokers with diabetes, NHB females, and those who did not graduate from high school. Health care providers may consider using objective measures of tobacco use, especially among those subgroups that are more likely to deny smoking, followed by tobacco cessation counseling and intervention information. Studies collecting self-reported smoking data must address the potentially invalid reporting of non-smoking, and should include a statement about the potentially biased estimate.

## Competing interests

The author(s) declare that they have no competing interests.

## Authors' contributions

MAF conceived of the study, completed the analyses, interpreted findings and led the writing. GWT, BJS, and SMD helped conceptualize ideas, interpret findings, and reviewed drafts of the manuscript. All authors read and approved the final manuscript.

## Pre-publication history

The pre-publication history for this paper can be accessed here:



## References

[B1] U.S. Department of Health and Human Services (2004). The health consequences of smoking: a report of the Surgeon General.

[B2] Khot UN, Khot MB, Bajzer CT, Sapp SK, Ohman EM, Brener SJ, Ellis SG, Lincoff AM, Topol EJ (2003). Prevalence of conventional risk factors in patients with coronary heart disease. JAMA.

[B3] August P (2003). Initial treatment of hypertension. N Engl J Med.

[B4] U.S. Department of Health and Human Services (1983). The health consequences of smoking: Cardiovascular diseases: a report of the Surgeon General.

[B5] Rimm EB, Chan J, Stampfer MJ, Colditz GA, Willett WC (1995). Prospective study of cigarette smoking, alcohol use, and the risk of diabetes in men. BMJ.

[B6] Manson JE, Ajani UA, Liu S, Nathan DM, Hennekens CH (2000). A prospective study of cigarette smoking and the incidence of diabetes mellitus among US male physicians. Am J Med.

[B7] Hu FB, Manson JE, Stampfer MJ, Colditz G, Liu S, Solomon CG, Willett WC (2001). Diet, lifestyle, and the risk of type 2 diabetes mellitus in women. N Engl J Med.

[B8] Sargeant LA, Khaw KT, Bingham S, Day NE, Luben RN, Oakes S, Welch A, Wareham NJ (2001). Cigarette smoking and glycaemia: the EPIC-Norfolk Study. Int J Epidemiol.

[B9] Wannamethee SG, Shaper A, Perry IJ (2001). Smoking as a modifiable risk factor for type 2 diabetes in middle-aged men. Diabetes Care.

[B10] Will JC, Galuska DA, Ford ES, Mokdad A, Calle EE (2001). Cigarette smoking and diabetes mellitus: evidence of a positive association from a large prospective cohort study. Int J Epidemiol.

[B11] Al-Delaimy WK, Manson JE, Solomon CG, Kawachi I, Stampfer MJ, Willett WC, Hu FB (2002). Smoking and risk of coronary heart disease among women with type 2 diabetes mellitus. Arch Intern Med.

[B12] Foy CG, Bell RA, Farmer DF, Goff DC, Wagenknecht LE (2005). Smoking and incidence of diabetes among U.S. adults. Diabetes Care.

[B13] Patja K, Jousilahti P, Hu G, Valle T, Qiao Q, Tuomilehto J (2005). Effects of smoking, obesity and physical activity on the risk of type 2 diabetes in middle-aged Finnish men and women. J Intern Med.

[B14] Tenenbaum A, Fisman EZ, Adler Y, Motro M, Boyko V, Behar S (2005). Smoking and development of type 2 diabetes in patients with decreased functional capacity. Int J Cardiol.

[B15] Howard G, Wagenknecht LE, Burke GL, Diez-Roux A, Evans GW, McGovern P, Nieto J, Tell GS (1998). Cigarette smoking and progression of atherosclerosis. JAMA.

[B16] Pardell H, Rodicio JL (2005). High blood pressure, smoking and cardiovascular risk. J Hypertens.

[B17] American Diabetes Association (2004). Standards of medical care in diabetes. Diabetes Care.

[B18] Wen CP, Cheng TY, Tsai SP, Hsu HL, Chan HT, Hsu CC (2006). Exploring the relationships between diabetes and smoking: with the development of "glucose equivalent" concept for diabetes management. Diabetes Res Clin Pract.

[B19] American Diabetes Association (2004). Smoking and diabetes. Diabetes Care.

[B20] US Department of Health and Human Services (1990). The health benefits of smoking cessation: a report of the Surgeon General.

[B21] Collins R, Peto R, MacMahon S, Hebert P, Fiebach NH, Eberlein KA, Godwin J, Qizilbash N, Taylor JO, Hennekens CH (1990). Blood pressure, stroke, and coronary heart disease. II: short-term reductions in blood pressure: overview of randomised drug trials in their epidemiological context. Lancet.

[B22] Gavin JR, Peterson K, Warren-Boulton E (2003). Reducing cardiovascular disease risk in patients with type 2 diabetes: A message from the National Diabetes Education Program. Am Fam Physician.

[B23] Hanna IR, Wenger NK (2005). Secondary prevention of coronary heart disease in elderly patients. Am Fam Physician.

[B24] Malarcher AM, Ford ES, Nelson DE, Chrismon JH, Mowery P, Merritt RK, Herman WH (1995). Trends in cigarette smoking and physicians' advice to quit smoking among people with diabetes in the U.S. Diabetes Care.

[B25] Kirk JK, Bell RA, Bertoni AG, Arcury TA, Quandt SA, Goff DC, Narayan KMV (2005). A qualitative review of studies of diabetes preventive care among minority patients in the United States, 1993–2003. Am J Manag Care.

[B26] Gillum RF (2005). Frequency of attendance at religious services and cigarette smoking in American women and men: The Third National Health and Nutrition Examination Survey. Prev Med.

[B27] Bramer SL, Kallungal BA (2003). Clinical considerations in study designs that use cotinine as a biomarker. Biomarkers.

[B28] Tunstall-Pedoe H, Woodward M, Brown CA (1991). The drinking, passive smoking, smoking deception and serum cotinine in the Scottish Heart Health Study. J Clin Epidemiol.

[B29] Pirkle JL, Flegal KM, Bernert JT, Brody DJ, Etzel RA, Maurer KR (1996). Exposure of the US population to environmental tobacco smoke. JAMA.

[B30] Bernert JT, Turner WE, Pirkle JL, Sosnoff CS, Akins JR, Waldrep MK, Ann Q, Covey TR, Whitfield WE, Gunter EW, Miller BB, Patterson DG, Needham LL, Hannon WH, Sampson EJ (1997). Development and validation of sensitive method for determination of serum cotinine in smokers and nonsmokers by liquid chromatography/atmospheric pressure ionization tandem mass spectrometry. Clinical Chemistry.

[B31] Stram DO, Huberman M, Wu AH (2002). Is residual confounding a reasonable explanation for the apparent protective effects of beta-carotene found in epidemiologic studies of lung cancer in smokers?. Am J Epidemiol.

[B32] Hatsukami DK, Benowitz NL, Rennard SI, Oncken C, Hecht SS (2006). Biomarkers to assess the utilitiy of potential reduced exposure tobacco products. Nicotine Tob Res.

[B33] Wei W, Kim Y, Boudreau N (2001). Association of smoking with serum and dietary levels of antioxidants in adults: NHANES III, 1988–1994. Am J Public Health.

[B34] Martinez ME, Reid M, Jiang R, Einspahr J, Alberts DS (2004). Accuracy of self-reported smoking status among participants in a chemoprevention trial. Prev Med.

[B35] McDonald SD, Perkins SL, Walker MC (2005). Correlation between self-reported smoking status and serum cotinine during pregnancy. Addict Behav.

[B36] Patrick DL, Cheadle A, Thompson DC, Diehr P, Koepsell T, Kinne S (1994). The validity of self-reported smoking: a review and meta-analysis. Am J Public Health.

[B37] Society for Research on Nicotine and Tobacco Subcommittee on Biochemical Verification (2002). Biochemical verification of tobacco use and cessation. Nicotine Tob Res.

[B38] Holl R, Grabert M, Heinze E, Debatin KM (1998). Objective assessment of smoking habits by urinary cotinine measurement in adolescents and young adults with type 1 diabetes. Diabetes Care.

[B39] Gariti P, Rosenthal DI, Lindell K, Hansen-Flaschen J, Shrager J, Lipkin C, Alterman AI, Kaiser LR (2002). Validating a dispstick method for detecting recent smoking. Cancer Epidemiol Biomarkers Prev.

[B40] Jones EE, Sigall H (1971). The bogus pipeline: A new paradigm for measuring affect and attitude. Psychol Bull.

[B41] Clark PI, Gautam SP, Hlaing WM, Gerson LW (1996). Response error in self-reported current smoking frequency by black and white established smokers. Ann Epidemiol.

[B42] Caraballo RS, Giovino GA, Pechacek TF (2004). Self-reported cigarette smoking vs serum cotinine among U.S. adolescents. Nicotine Tob Res.

[B43] Suadicani P, Hein HO, Gyntelberg F (1997). Mortality and morbidity of potentially misclassified smokers. Int J Epidemiol.

[B44] Vartiainen E, Seppala T, Lillsunde P, Puska P (2002). Validation of self reported smoking by serum cotinine measurement in a community-based study. J Epidemiol Community Health.

[B45] Wells AJ, English PB, Posner SF, Wagenknecht LE, Perez-Stable EJ (1998). Misclassification rates for current smokers misclassfied as nonsmokers. Am J Public Health.

[B46] National Center for Health Statistics: National Health and Nutrition Examination Survey III Documentation, Codebook, and Data Files. http://www.cdc.gov/nchs/about/major/nhanes/datalink.htm#NHANESIII.

[B47] Jarvis MJ, Tunstall-Pedoe H, Feyerabend C, Vesey C, Saloojee Y (1987). Compariston of tests used to distinguish smokers from nonsmokers. Am J Public Health.

[B48] Hyman DJ, Pavlik VN (2001). Characteristics of patients with uncontrolled hypertension in the United States. N Engl J Med.

[B49] Borrell LN, Burt BA, Neighbors HW, Taylor GW (2004). Social factors and periodontitis in an older population. Am J Public Health.

[B50] Mainous AG, King DE, Garr DR, Pearson WS (2004). Race, rural residence, and control of diabetes and hypertension. Ann Fam Med.

[B51] Fiore MC, Bailey WC, Cohen SJ, Dorfman SF, Goldstein MG, Gritz ER, Heyman RB, Jaén CR, Kottke TE, Lando HA, Mecklenburg RE, Mullen PD, Nett LM, Robinson L, Stitzer ML, Tommasello AC, Villejo L, Wewers ME (2000). Treating Tobacco Use and Dependence. Clinical Practice Guideline.

[B52] (2005). SUDAAN release 901, Software for the Statistical Analysis of Correlated Data, Research Triangle Park, NC: Research Triangle Institute.

[B53] Twardella D, Loew M, Rothenbacher D, Stegmaier C, Ziegler H, Brenner H (2006). The diagnosis of smoking related-disease is a prominent trigger for smoking cessation in a retrospective cohort study. J Clin Epidemiol.

[B54] Ford ES, Malarcher AM, Herman WH, Aubert RE (1994). Diabetes mellitus and cigarette smoking. Findings from the 1989 National Health Interview Survey. Diabetes Care.

[B55] Caraballo RS, Giovino GA, Pechacek TF, Mowery PD (2001). Factors associated with discrepancies between self-reports on cigarette smoking and measured serum cotinine levels among persons aged 17 years or older: Third National Health and Nutrition Examination Survey, 1988–1994. Am J Epidemiol.

[B56] Alamar B, Glantz SA (2006). Effect of increased social unacceptability of cigarette smoking on reduction in cigarette consumption. Am J Public Health.

[B57] U.S. Department of Health and Human Services (2000). With Understanding and Improving Health and Objectives for Improving Health. Healthy People 2010.

[B58] Centers for Disease Control and Prevention (2005). Cigarette smoking among adults – United States, 2004. MMWR.

[B59] Guo H, Eskridge KM, Christensen D, Qu M, Safranek T (2002). Statistical adjustment of lying about seat belt and alcohol use with motor vehicle accident data. Proceedings of the American Statistical Association.

[B60] Lyles RH, Allen AS, Flanders WD, Kupper LL, Christensen DL (2006). Inference for case-control studies when exposure status is both informatively missing and misclassified. Stat Med.

